# Effect of oxytocin and cloprostenol on the seminal parameters of red brocket deer (*Mazama rufa*) during the electroejaculation procedure

**DOI:** 10.1590/1984-3143-AR2024-0074

**Published:** 2025-03-24

**Authors:** Cláudia Maria Herédias-Ribas, Yuki Tanaka, José Maurício Barbanti Duarte

**Affiliations:** 1 Laboratório das Biotecnologias da Reprodução, Departamento de Patologia, Reprodução e Saúde Única, Faculdade de Ciências Agrárias e Veterinárias, Universidade Estadual Paulista, Jaboticabal, SP, Brasil; 2 Núcleo de Pesquisa e Conservação de Cervídeos, Departamento de Zootecnia, Faculdade de Ciências Agrárias e Veterinárias, Universidade Estadual Paulista, Jaboticabal, SP, Brasil

**Keywords:** Cervidae, hormonal stimulation, red brocket deer, reproductive biotechnologies, semen collection

## Abstract

Successful implementation of conservation programs for endangered species requires biological material for use in reproductive biotechnologies. This enhances reproductive efficiency and helps increase the populations of critically endangered species. One way to facilitate the exchange of genetics between captive and free-ranging animals is through the creation of cryogenic banks that store cryopreserved gametes. In particular, semen cryopreservation allows for this exchange to occur. We evaluated whether the use of exogenous hormones (such as oxytocin and prostaglandin) prior to electroejaculation increases seminal volume, sperm concentration, and the number of doses produced in the red brocket deer (*Mazama rufa*). We also evaluate whether seminal parameters vary over the three stimulation cycles of the same electroejaculation procedure. The treatments did not affect ejaculate volume (p = 0.402), the number of sperm cells in the ejaculates (p = 0.926), total doses produced (p = 0.684), sperm mass movement (p = 0.229), sperm cell concentration (p = 0.106), and acrosome integrity (p = 0.210). The use of hormones has potential in reducing the need for stressful stimuli in electroejaculation, but the choice of hormones must take into account their effects on semen quality.

## Introduction

Many cervid populations have experienced a significant decline attributed to several factors, including habitat loss, diseases transmitted by domestic ungulates, and predation by feral dogs (Duarte and Reis, 2012). This population decline leads to diminished genetic diversity because of genetic drift, which combined with inbreeding depression, accelerates the process known as the extinction vortex (Frankham, 2008).

Reproductive biotechnologies are effective tools for intervening in the process of genetic loss, enabling the preservation of populations in cryogenic banks (Rola et al., 2021). Among these techniques, semen cryopreservation is particularly important because it facilitates the transfer of genetic material between institutions and zoos. This approach eliminates challenges associated with animal transportation, reduces expenses, minimizes animal stress, mitigates incompatibility issues between individuals, and reduces the risk of injury as well as disease transmission (Pukazhenthi and Wildt, 2004). Semen collection can be performed using various techniques, such as the use of an artificial vagina, electroejaculation, epididymal collection, and testicular biopsy (Bainbridge and Jabbour, 1998; Silva et al., 2004; Spindler and Wildt, 2010; Silva et al., 2012; Silva et al., 2015). For wildlife, electroejaculation has been the preferred technique because it can be performed under anesthesia, thus eliminating the need for training or habituation to handling (Spindler and Wildt, 2010).

Studies in domestic animals have shown that semen volume and sperm count in ejaculates increase after the intravenous administration of oxytocin five minutes before semen collection (Knight and Lindsay, 1970; Berndtson and Igboeli, 1988; Assinder et al., 2000). This increase occurs because oxytocin stimulates the smooth muscle of the epididymis, thereby facilitating the transport of sperm through the ducts before and during ejaculation (Knight, 1974; Berndtson and Igboeli, 1988). Prostaglandin F2α (PGF) also increases smooth muscle contractility in both males and females, which may facilitate the ejaculatory process. This effect has been demonstrated in dogs, stallions, buffalo, and cattle (Cornwell et al., 1974; Hess, 2002; Ibrahim, 1988; Palmer et al., 2004).

This study evaluates whether oxytocin and cloprostenol (a synthetic analog of prostaglandin F2α) increase sperm concentration and semen volume in the red brocket deer (*Mazama rufa*) without affecting parameters of semen quality.

## METHODS

This study was approved by the Ethics Committee on the Use of Animals (CEUA) of the Faculdade de Ciências Agrárias e Veterinárias (FCAV) of the Universidade Estadual Paulista (UNESP), Jaboticabal, São Paulo, Brazil (approval number 4620/2018), in accordance with the ethical principles adopted by the Colégio Brasileiro de Experimentação Animal (COBEA).

### Animals

We used four adult male red brocket deer (*Mazama rufa*), all healthy and of reproductive age (1–5 years old; body mass = 35.2 ± 3.5 kg). These deer were part of the herd at the Núcleo de Pesquisa e Conservação de Cervídeos of the Faculdade de Ciências Agrárias e Veterinárias, Universidade Estadual Paulista, Jaboticabal. Animals were housed individually in 12 m^2^ (3 × 4 m) stalls, allowing olfactory and auditory contact with conspecifics and exposure to natural light. The deer had access to water *ad libitum* and were fed daily. Their diet consisted of pelleted feed (Equitech®, Presence, Paulínia, São Paulo, Brazil) and approximately 1 kg/animal/day of perennial soybean (*Neonotonia wightii*), ramie (*Boehmeria nivea*), or fresh mulberry (*Morus alba*) branches, depending on availability in the field.

### Treatments

**Figure 1:**
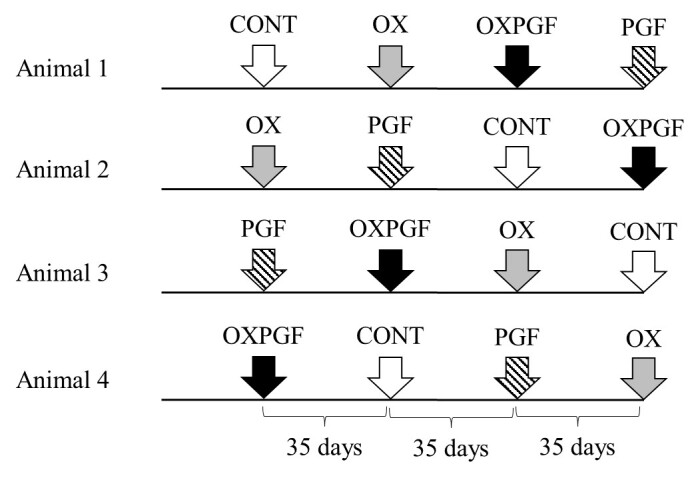
Diagram of the experimental model of the four hormonal treatments to which male *Mazama rufa* were subjected. The interval between collections was 35 days. CONT: control group (0.5 mL of 0.9% saline solution); OX: oxytocin (20 UI); OXPGF: combination of oxytocin (20 UI) and cloprostenol (0.25 mg); PGF: cloprostenol (0.25 mg).

We conducted four hormone treatments using a Latin square experimental design. This design ensured that the treatment sequence was not repeated for any two animals or within the same harvest period, thus eliminating the effects of treatment sequence and individual variation (Figure 1).

**Figure 2:**
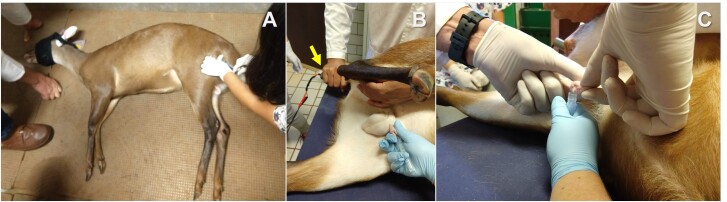
Electroejaculation procedure in red brocket deer (*Mazama rufa*). **A.** Treatment administered by intramuscular injection after the loss of consciousness induced by chemical restraint. **B.** The electroejaculation procedure in progress. Note that the electroejaculation probe (yellow arrow) is inserted into the animal's rectum, while the conical collection tube is positioned at the animal's prepuce. **C.** Semen collection following electroejaculation stimulation. Photos: NUPECCE.

Treatments were administered after the loss of consciousness induced by chemical restraint (see below, Figure 2A). The treatments included: (1) 0.5 ml of 0.9% saline solution as the control (CONT) group, (2) 20 IU of oxytocin (hereafter OX; Ocitocina Forte, UCBVet Saúde Animal, Jaboticabal, SP, Brazil;), (3) 0.25 mg of cloprostenol (hereafter PGF; synthetic prostaglandin, Ciosin®, MSD Saúde Animal, São Paulo, SP, Brazil;), and (4) 20 IU of oxytocin + 0.25 mg of cloprostenol (hereafter OXPGF). Semen was collected at 35-day intervals, with the first collection serving to empty the reproductive tract.

### Eletroejaculation

For semen collection, the animals were chemically restrained with an intramuscular administration of 1 mg/kg xylazine and 7 mg/kg ketamine. The animals were positioned in lateral decubitus, and their rectums were emptied to prevent fecal matter from disrupting the conduction of electrical stimuli. Moreover, the prepuce was washed with a 0.9% saline solution.

Collection by electroejaculation was conducted as described by Favoretto et al. (2012) for cervids, with some modifications. Each animal received 10 electrical stimuli, with intensities ranging from 250 to 750 mA (P.T. Electronics, Boring, OR, USA). Each stimulus lasted 3 seconds, with a 3-second rest period between stimuli. Before initiating the stimuli, we checked for semen in the prepuce by spontaneous ejaculation (T0). Next, three cycles (C1, C2, and C3) of 10 stimuli were performed, with 5-minute intervals between cycles. Semen was collected in conical tubes preheated in a water bath at 37 ^o^C (Figure 2). The collection tube was replaced after each electrostimulation cycle and kept in the water bath (37 ^o^C) until evaluation. The evaluations were performed independently to determine whether seminal parameters changed during the electroejaculation procedure.

### Seminal evaluation

Semen was evaluated for physical and morphological characteristics following the guidelines of the Colégio Brasileiro de Experimentação Animal (CBRA, 2013). All evaluations were performed by a single evaluator who was unaware of the treatment administered (double-blind), thus eliminating evaluator bias. Each ejaculate (undiluted fresh semen) was evaluated for volume (using an automatic micropipette, in µL), color (whitish, white, ivory, yellow, or other), appearance (creamy, milky, or watery), pH (using a Merck reagent strip, Darmstadt, Germany), and wave motion (mass movement of sperm, rated from 0 to 5).

Because the ejaculates of *Mazama* spp. contain a high concentration of sperm (1,100-3,995 × 10^6^ sperm/mL for *M. americana*; Favoretto et al., 2012), we initially diluted the semen 1:2 with Tris-yolk (a solution comprising Tris buffer, glycerol, citric acid, glucose, distilled water, and egg yolk). We conducted the microscopic evaluation subjectively, replicating the procedure used in free-ranging animals. We used an Olympus CX31 optical microscope (Olympus Corporation) to evaluate four seminal parameters: (1) vigor (the intensity of sperm flagellar movement on a scale from 0 to 5), (2) motility total (the proportion of motile sperm, measured in %), (3) acrosome integrity (using simple acrosome staining; Pope et al., 1991), and (4) plasma membrane integrity (using the eosin-nigrosin staining method).

Sperm concentration was evaluated using a Neubauer hemocytometer chamber by analyzing an aliquot of fresh semen (without any dilution) fixed in formalin saline (1:200 dilution). This allowed us to calculate the concentration of sperm per milliliter of ejaculate (sptz × 10^9^/mL) as well as the concentration of sperm in each individual ejaculate. The number of inseminating doses collected was determined after accounting for the amount of diluent (Tris-yolk), based on the sperm concentration and the volume of the ejaculate. The doses were filled into 0.25 mL straws, each containing 25 × 10^6^ sperm.

### Data analysis

To evaluate the effects of the treatments (CONT, OX, PGF, and OXPGF) on total seminal parameters, we summed the values from each cycle of electroejaculation stimulation for each animal (T0 + C1 + C2 + C3). To check the effects of the treatments on the seminal parameters of the individual ejaculates (T0, C1, C2, C3), we used the raw values obtained in each stimulation cycle. The results are presented using descriptive statistics (mean and standard error) in the R software (R Development Core Team, 2013). We checked the normality of the residuals using the Shapiro-Wilk test and the homogeneity of variances using Bartlett's test. We performed the *F*-test for parametric data and the Tukey test for nonparametric data. The significance level for all tests was set at 5%.

## Results

Ejaculates were obtained in all cycles of electroejaculation stimulation. The exceptions were two animals in the control group; one animal ejaculated only during the third stimulation cycle, and the other did not ejaculate during the third cycle (Table 1). In four semen collection procedures (2 CONT, 1 PGF, and 1 OX), ejaculation occurred prior to the electroejaculation procedure (T0).

**Table 1:** Mean and standard error (SE) of the physicochemical (volume and pH) and microscopic characteristics (mass movement, motility, vigor and concentration) of semen from different red brocket deer (*Mazama rufa*) ejaculates collected by electroejaculation under the influence of 0.9% saline solution (control group, CONT), 20 IU of oxytocin (OX), 0.25mg of cloprostenol (PGF), or a combination of oxytocin and cloprostenol (OXPGF).

	**Control (CONT)**				**Oxytocin (OX)**				**Cloprostenol (PGF)**				**Association (OXPGF)**			
	**T0**	**C1**	**C2**	**C3**	**T0**	**C1**	**C2**	**C3**	**T0** *	**C1**	**C2**	**C3**	T0*	**C1**	**C2**	**C3**
**Total volume (mL)**	0.900 ± 0.02	0.560 ± 0.12	0.225 ± 0.05	0.515 ± 0.12	0.040 ± 0.00	0.205 ± 0.04	0.925 ± 0.17	1.380 ± 0.17	0.04	0.600 ± 0.11	0.625 ± 0.17	0.380 ± 0.09	0.035	1.010 ± 0.15	0.249 ± 0.09	0.612 ± 0.18
**pH**	6.25 ± 1.77	6.83 ± 0.29	7.0 ± 0.50	7.5 ± 0.00	7.0 ± 0.24	7.50 ± 0.91	7.88 ± 0.29	8.25 ± 0.29	7.00	7.38 ± 0.85	8.0 ± 0.91	8.0 ± 0.82	7.00	7.0 ± 0.41	7.38 ± 0.75	8.25 ± 0.87
**Mass movement (0-5)**	4.0 ± 0.00^ab^	5.0 ± 0.00^a^	4.0 ± 0.00^ab^	2.67 ± 0.58^b^	3.0 ± 0.95	4.0 ± 0.82	2.75 ± 1.50	3.25 ± 1.50	2.00	3.75 ± 1.89	3.0 ± 1.29	2.50 ± 1.29	4.00^ab^	4.25 ± 0.50^a^	2.50 ± 1.29^ab^	2.25 ± 0.96^b^
**Vigor (0-5)**	3.5 ± 0.71	3.67 ± 0.58	3.0 ± 0.00	2.67 ± 0.58	3.0 ± 0.33	3.50 ± 0.58	3.0 ± 0.50	3.25 ± 0.50	2.00	3.0 ± 1.15	2.75 ± 0.96	2.33 ± 0.58	3.00	2.50 ± 0.58	2.33 ± 0.58	2.25 ± 0.50
**Motility (%)**	90.0 ± 0.00^a^	86.67 ± 5.77^a^	75.0 ± 5.00^ab^	70.0 ± 0.00^b^	80.0 ± 6.09	85.0 ± 10.00	81.67 ± 16.52	68.75 ± 16.52	30.00	72.50 ± 23.63	60.0 ± 19.15	56.67 ± 20.82	80.00	77.50 ± 9.57	60.0 ± 20.82	42.50 ± 33.04
**Concentration (×10^9^/mL)**	5.07 ± 2.73	5.06 ± 1.05	3.81 ± 1.83	3.40 ± 1.21	2.90 ± 0.00^ab^	5.48 ± 2.46^a^	2.71 ± 0.61^ab^	1.19 ± 0.61^b^	0.66	4.25 ± 2.95	1.23 ± 1.29	1.47 ± 1.70	5.70	5.73 ± 2.58	4.09 ± 3.15	0.83 ± 0.13
**Sperm/ejaculate (×10^9^)**	0.26 ± 0.23	1.01 ± 0.70	0.22 ± 0.03	0.70 ± 0.65	0.12 ± 0.00	0.22 ± 0.19	0.48 ± 0.23	0.36 ± 0.23	0.00	0.79 ± 0.72	0.39 ± 0.58	0.30 ± 0.37	0.20	1.12 ± 1.43	0.39 ± 0.07	0.18 ± 0.19
**Total doses (n)**	21.0 ± 9.19	121.0 ± 27.93	26.0 ± 1.53	82.0 ± 25.79	5.0 ± 5.07	34.0 ± 3.06	75.0 ± 9.33	58.0 ± 9.33	1.00	126.00 ± 28.87	23.0 ± 10.50	35.0 ± 15.04	8.00	223.0 ± 49.26	46.0 ± 3.06	20.0 ± 8.33
**Motile cells in the ejaculate (×10^9^)**	0.23 ± 0.21	0.90 ± 0.64	0.17 ± 0.03	12.36 ± 20.47	0.09 ± 0.00	0.20 ± 0.17	0.51 ± 0.20	0.26 ± 0.20	0.00	0.68 ± 0.66	0.31 ± 0.483	0.22 ± 0.31	0.16	0.80 ± 0.99	0.27 ± 0.07	0.33 ± 0.54
**Membrane integrity (%)**	92.25 ± 3.89	93.0 ± 3.981	83.5 ± 8.89	59.5 ± 45.55	92.0 ± 1.02	91.88 ± 6.14	93.67 ± 2.50	92.25 ± 2.50	93.00	70.38 ± 43.60	68.88 ± 45.92	61.17 ± 53.14	97.50	82.25 ± 23.20	82.67 ± 8.05	75.13 ± 35.57
**Acrosome** **integrity (%)**	96.5 ± 2.12	91.5 ± 3.91	88.33 ± 5.80	67.17 ± 45.55	86.0 ± 1.42^ab^	83.67 ± 11.25^b^	85.38 ± 2.17^b^	96.88 ± 2.17^a^	89.00	84.88 ± 13.85	92.25 ± 4.29	92.33 ± 0.71	84.00	84.25 ± 14.36	83.67 ± 14.78	86.67 ± 5.89

* Only one animal ejaculed before the first cycle of electroejaculation.

All the collected samples were classified as white, and their appearance varied among creamy (CONT: 66.67 ± 0.41%, n = 08; OX: 53.85 ± 0.48%, n = 07; PGF: 33.33 ± 0%, n = 04; OXPGF: 46.15 ± 0.50%, n = 06), milky (CONT: 8.33 ± 0%, n = 01; OX: 23.08 ± 0.35%, n = 03; PGF: 33.33 ± 0.29%, n = 04; OXPGF: 30.77 ± 71.00%, n = 04), and aqueous (CONT: 25.00 ± 0.35%, n = 03; OX: 23.08 ± 0%, n = 03; PGF: 33.33 ± 0.29%, n = 04; OXPGF: 30.77 ± 0.71%, n = 04). This variation indicates different proportions of seminal plasma and sperm cells in the samples, with the creamy appearance indicating a higher concentration of cells. However, treatment did not influence sample appearance (p = 0.702).

**Figure 3:**
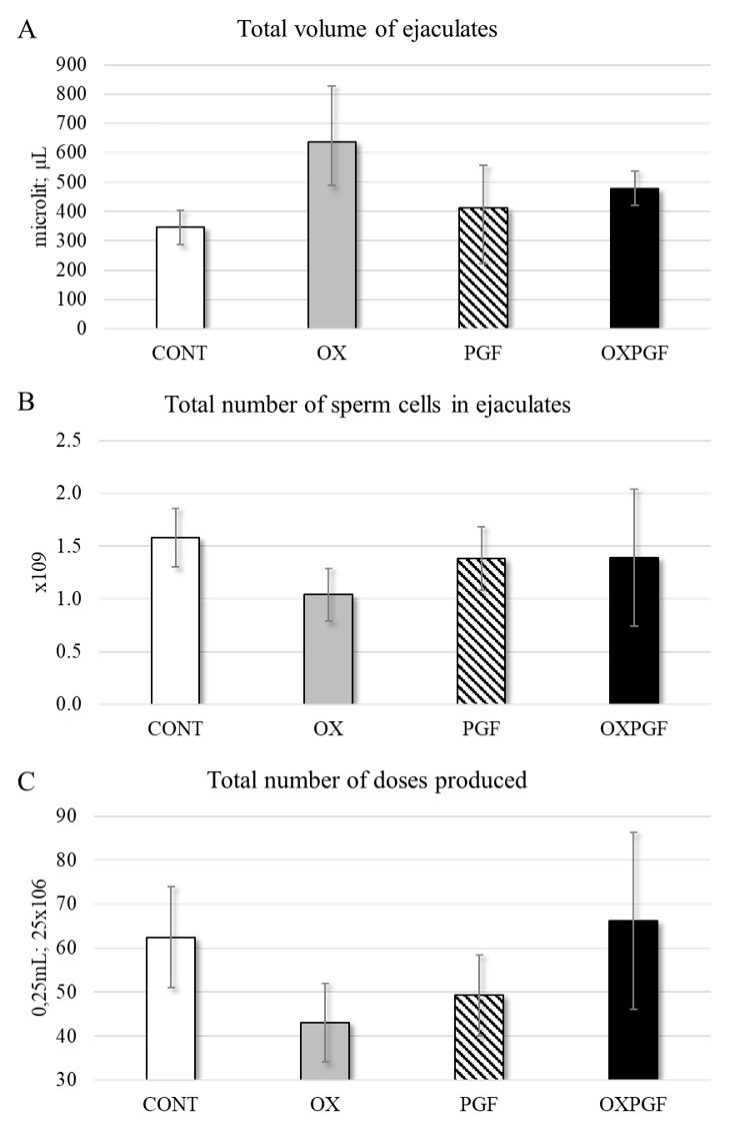
Graphical representation of sperm evaluation (mean ± SE) of *Mazama rufa*. **A.** total ejaculate volume (µL). **B.** Number of sperm in the ejaculate (×10^9^). **C.** Number of doses produced (0.25 mL straws with an insemination dose of 25 ×10^6^).

When comparing the ejaculates (Figure 3), we found no effect of treatment on the total ejaculate volume (p = 0.402) and on the number of cells in the ejaculates (p = 0.926), and thus no effect on the number of doses produced (p = 0.684). Moreover, we found no effect of treatment on wave motion (p = 0.229), sperm cell concentration (p = 0.106), and acrosome integrity (p = 0.210).

The treatments affected the pH of the semen (p = 0.004), sperm vigor (p = 0.0002), sperm motility (p = 0.003), and the integrity of the plasma membrane (p = 0.006). The mean pH of the ejaculates did not differ between the treated groups but was consistently higher than the pH of the control group. The exception was the OXPGF treatment, which did not differ from the control group.

Sperm vigor in the OXPGF group was lower than that of the other groups, but it did not differ significantly from the PGF group. Although the vigor in the OX group did not differ significantly from that of the control group, it showed the highest values.

Motility was negatively affected in the animals that received cloprostenol (PGF group) and the combination of hormones (OXPGF group). However, oxytocin (OX group) did not affect motility, as the values were similar to those in the control group. Although motility was negatively affected in the OXPGF group, it did not differ significantly from the group that received oxytocin.

The animals in the OX and OXPGF groups exhibited a higher proportion of sperm with an intact plasma membrane, although these proportions were not significantly different from those in the control group. The animals in the OX and OXPGF groups exhibited a higher proportion of sperm with intact plasma membranes, although these proportions did not differ from the control group. The animals in the PGF group exhibited the lowest number of cells with intact membranes, although these values did not differ from the OXPGF and control groups.

During the collection of semen from cervids using electroejaculation, it is unnecessary to complete all three cycles of electrostimulation. Once the appearance of the semen changes from milky to watery, which indicates a depletion of stored semen, the stimulation process is interrupted. Based on this, we also examined the effect of hormones throughout the procedure of semen collection. We found that treatment with OXPFG influenced the mass movement of sperm cells within the same electrostimulation cycle (p = 0.026). The highest mass movement was observed in the samples from the first stimulation cycle (C1), and the lowest mass movement was observed in the samples from the third cycle (C3). However, the mass movement in cycles C1 and C3 was similar to that in the second cycle (C2) and to that of the semen collected before starting the stimulation (T0).

The proportion of motile cells in the ejaculate of the control group also varied significantly (p = 0.006). The proportion was highest in the first post-stimulus collection (C1), but it only differed from the third cycle (C3), which had the lowest proportion. However, the third cycle was statistically similar to the second cycle (C2). The number of inseminating doses produced in the OXPFG group varied significantly between collection times (p = 0.044). The first stimulation cycle (C1) produced the highest number of doses, and the second cycle (C2) produced the lowest number of doses. The number of inseminating doses produced was similar among the other cycles.

The concentration of sperm cells (p = 0.040) and the proportion of sperm cells with intact acrosomes (p = 0.018) differed between the collection times in the OX group. In terms of sperm cell concentration, the highest concentration was observed in the first cycle (C1), which was significantly different from the lowest concentration in the third cycle (C3). However, there were no significant differences between the other times (T0 and C2). Regarding acrosome integrity, the highest proportion of cells with intact acrosomes was observed in the third cycle (C3), which differed significantly from the lowest proportions observed in the first and second cycles (C1 and C2).

Therefore, when there is an effect of collection time within each treatment, the first collection cycle yields the best results. However, when examining the effects of treatments within a single collection time, no significant treatment effects were observed for any of the variables (p > 0.05).

## Discussion

The red brocket deer (*Mazama rufa*) was recently revalidated as a species by Peres et al. (2021). This species was previously considered synonymous with *Mazama americana*, which is considered a polyphyletic complex of cryptic species with large chromosomal divergence that results in reproductive isolation (Cursino et al., 2014; Salviano et al. 2017). Thus, this study is the first to present seminal characteristics of *Mazama rufa*.

The mean ejaculate volume (347.50 ± 54.94 µL, CONT) we obtained in *M. rufa* was similar to that obtained for *M. americana* using the same technique (320 ± 160 µL in Favoretto et al., 2012; 390 ± 140 µL in Rola et al., 2013; 200–400 µL in Duarte and Garcia, 1995).

Although we observed an effect of the treatments on semen pH, the values observed are within the pH range (6 to 8) for other cervids (Rola et al., 2013), but higher than those reported for cattle (6.4 to 7.8) and sheep (5.9 to 7.3) (Garner and Hafez, 2004).

Previous studies have primarily examined the effects of exogenous oxytocin and prostaglandin on electroejaculation in cattle and sheep, focusing on whether these hormones reduce the duration of the electroejaculation procedure, especially the time and number of stimuli required for ejaculation (Palmer et al., 2004; Ungerfeld et al., 2016; Ungerfeld et al., 2018; Fernandes et al., 2021). The use of the electroejaculator can cause stress and pain for the animal, thus raising concerns about animal welfare. It can also alter physiological patterns, such as serum cortisol concentration, respiratory and heart rate, as well as hematological and biochemical parameters (Stafford et al., 1996; Mosure et al., 1998; Abril‐Sánchez et al., 2017). This is particularly true for cervids, which are highly susceptible to stressful stimuli (Duarte, 2010).

Although we found no significant differences between the treatments and the control group, or in relation to the ejaculates or collection time, we believe that some form of effect occurred. This idea is supported by the higher seminal volume collected in the treatments with exogenous hormones compared to the control group and *M. americana* (Duarte and Garcia, 1995; Favoretto et al., 2012; Rola et al., 2013). This is because both hormones can stimulate ejaculatory processes. Prostaglandin promotes peristaltic contractions of the seminiferous tubules, which may favor the exit of sperm from the Sertoli cells, moving them towards the rete testis and eventually the epididymides (Ellis et al., 1981). Oxytocin is associated with penile erection, and its hypothalamic pulse is associated with ejaculation (Thackare et al., 2006). Moreover, oxytocin acts directly on the activation of the myosin light chain, causing muscular contraction of the seminiferous tubules (Worley et al., 1956; Niemi and Kormano, 1965), the epididymides, the vas deferentia, and the ampulla (Nicholson et al., 1999; Whittington et al., 2001). Additionally, it indirectly stimulates the synthesis of prostaglandin F2α (Fuchs et al., 1981; Phaneuf et al., 1993).

Unlike previous studies that reported no influence of treatment on semen quality (Ungerfeld et al., 2016; Ungerfeld et al., 2018; Fernandes et al., 2021), we found negative effects on seminal quality in treatments using only prostaglandin. In this treatment, we observed the highest rates of sperm exhibiting plasma membrane damage. This could be attributed to the role of prostaglandin in selectively controlling the phospholipid membrane of sperm during capacitation, an important process in oocyte fertilization (Voglmayr, 1973; Kelly, 1981). The use of this protocol may have accelerated the process of sperm maturation and capacitation, making the sperm vulnerable to plasma membrane damage.

Most studies involving oxytocin and prostaglandin treatments in small ruminants aimed to determine whether the electroejaculation procedure could be shortened, thereby reducing the time the animals are exposed to the stressful stimuli of electroejaculation. However, these experiments were conducted with the animals awake and without the use of anesthesia (Ungerfeld et al., 2018; Fernandes et al., 2021). These studies found that the use of hormones reduced the number of stimuli needed for ejaculation. Ungerfeld et al. (2018) suggest that using hormones could facilitate semen collection in wild ruminants, such as cervids. This is relevant because these animals need chemical restraint to perform the procedure, exhibit an increased risk of mortality during prolonged anesthesia, and are highly sensitive to stressors (Duarte, 2010).

## Conclusion

Although we found no significant differences among the treatment groups and the control group in terms of ejaculate volume and sperm concentration in the collection cycles, we believe that the treatments may still have influenced certain seminal parameters. Our findings indicate that the first cycles of electrical stimulation improved semen quality parameters, suggesting that the electroejaculation procedure can be terminated once ejaculation occurs, thereby preserving semen volume and characteristics. Based on our findings on acrosome integrity, we advise against using the cloprostenol protocol in conjunction with the electroejaculation procedure in red brocket deer.

## References

[B001] Abril‐Sánchez S, Freitas‐de‐Melo A, Damián JP, Giriboni J, Villagrá‐García A, Ungerfeld R (2017). Ejaculation does not contribute to the stress response to electroejaculation in sheep. Reprod Domest Anim.

[B002] Assinder SJ, Carey M, Parkinson T, Nicholson HD (2000). Oxytocin and vasopressin expression in the ovine testis and epididymis: changes with the onset of spermatogenesis. Biol Reprod.

[B003] Bainbridge DRJ, Jabbour HN (1998). Potential of assisted breeding techniques for the conservation of endangered mammalian species in captivity: a review. Vet Rec.

[B004] Berndtson WE, Igboeli G (1988). Spermatogenesis, sperm output and seminal quality of Holstein bulls electroejaculated after administration of oxytocin. J Reprod Fertil.

[B005] CBRA (2013). Manual para exame andrológico e avaliação de sêmen animal..

[B006] Cornwell J, Koonce K, Kreider J (1974). Effect of prostaglandin F2α on seminal characteristics of the stallion. Anim Sci J.

[B007] Cursino MS, Salviano MB, Abril VV, Zanetti EDS, Duarte JMB (2014). The role of chromosome variation in the speciation of the red brocket deer complex: the study of reproductive isolation in females. BMC Evol Biol.

[B008] Duarte JMB, Garcia JM (1995). Reprodução assistida em Cervidae brasileiros. Rev Bras Reprod Anim.

[B009] Duarte JMB, Reis ML (2012). Plano de ação nacional para a conservação dos cervídeos ameaçados de extinção.

[B010] Duarte JMB, Duarte JMB, González S (2010). Neotropitcal cerviology: biology and medicine of Latin American deer..

[B011] Ellis LC, Farr GCH, Tesi RJ (1981). Contractility of seminiferous tubules as related to sperm transport in the male. Arch Androl.

[B012] Favoretto SM, Zanetti ES, Duarte JMB (2012). Cryopreservation of red brocket deer semen (*Mazama americana*): comparison between three extenders. J Zoo Wildl Med.

[B013] Fernandes DAM, Souza CV, Balaro MFA, Santos JDR, Santos VMB, Costa MMCP, Carvalho ABS, Rodrigues ALR, Ungerfeld R, Brandão FZ (2021). Response of rams to electroejaculation following the administration of oxytocin and cloprostenol with or without GnRH. Theriogenology.

[B014] Frankham R (2008). Genetic adaptation to captivity in species conservation programs. Mol Ecol.

[B015] Fuchs AR, Husslein P, Fuchs F (1981). Oxytocin and the initiation of human parturition: II. Stimulation of prostaglandin production in human decidua by oxytocin. Am J Obstet Gynecol.

[B016] Garner DL, Hafez ESE, Hafez ESE, Hafez B (2004). Reprodução animal..

[B017] Hess M (2002). The effects of prostaglandin-F2α, oxytocin and gonadotropin releasing hormone on ejaculate characteristics in the dog.

[B018] Ibrahim M (1988). Influence of oxytocin and prostaglandin on semen characteristics and process of ejaculation in buffalo bulls. Acta Vet Hung.

[B019] Kelly RW (1981). Prostaglandin synthesis in the male and female reproductive tract. Reprod..

[B020] Knight TW, Lindsay DR (1970). Short and long-term effects of oxytocin on quality and quantity of semen from rams. J Reprod Fertil.

[B021] Knight TW (1974). A qualitative study of the factors affecting the contractions of the epidicymis and ductus deferens of the ram. J Reprod Fertil.

[B022] Mosure WL, Meyer RA, Gudmundson J, Barth AD (1998). Evaluation of possible methods to reduce pain associated with electroejaculation in bulls. Can Vet J.

[B023] Nicholson HD, Parkinson TJ, Lapwood KR (1999). Effects of oxytocin and vasopressin on sperm transport from the cauda epididymis in sheep. J Reprod Fertil..

[B024] Niemi M, Kormano M (1965). Histochemical demonstration of a C-esterase activity in the seminiferous tubules of the rat testis. Reproduction.

[B025] Palmer CW, Amundson SD, Brito LFC, Waldner CL, Barth AD (2004). Use of oxytocin and cloprostenol to facilitate semen collection by electroejaculation or transrectal massage in bulls. Anim Reprod Sci.

[B026] Peres PH, Luduvério DJ, Bernegossi AM, Galindo DJ, Nascimento GB, Oliveira ML, Sandoval EDO, Vozdova M, Kubickova S, Cernohorska H, Duarte JMB (2021). Revalidation of *Mazama rufa* (Illiger 1815) (Artiodactyla: Cervidae) as a distinct species out of the complex *Mazama americana* (Erxleben 1777). Front Genet.

[B027] Phaneuf S, Europe-Finner GN, Varney M, Mackenzie IZ, Watson SP, Bernal AL (1993). Oxytocin-stimulated phosphoinositide hydrolysis in human myometrial cells: involvement of pertussis toxin-sensitive and-insensitive G-proteins. J Endocrinol.

[B028] Pope CE, Zhang YZ, Dresser BL (1991). A simple staining method for evaluating acrosomal status of cat spermatozoa. J Zoo Wildl Med.

[B029] Pukazhenthi BS, Wildt DE (2004). Which reproductive technologies are most relevant to studying, managing and conserving wildlife?. Reprod Fertil Dev.

[B030] R Development Core Team (2013). R: a language and environment for statistical computing..

[B031] Rola LD, Buzanskas ME, Melo LM, Chaves MS, Freitas VJF, Duarte JMB (2021). Assisted reproductive technology in neotropical deer: a model approach to preserving genetic diversity. Animals.

[B032] Rola LD, Zanetti ES, Duarte JMB (2013). Evaluation of semen characteristics of the species *Mazama americana* in captivity. Anim Prod Sci.

[B033] Salviano MB, Cursino MS, Zanetti EDS, Abril VV, Duarte JMB (2017). Intraspecific chromosome polymorphisms can lead to reproductive isolation and speciation: an example in red brocket deer (*Mazama americana*). Biol Reprod.

[B034] Silva AR, Lima GL, Peixoto GCX, Souza ALP (2015). Cryopreservation in mammalian conservation biology: current applications and potential utility. Res Rep Bio Stud..

[B035] Silva AR, Morato RG, Silva LDM (2004). The potential for gamete recovery from non-domestic canids and felids. Anim Reprod Sci.

[B036] Silva AR, Souza ALP, Santos EAAD, Lima GL, Peixoto GCX, Souza PC, Castelo TS (2012). Formação de Bancos de Germoplasma e sua contribuição para a conservação de espécies silvestres no Brasil. Ciênc Anim.

[B037] Spindler RE, Wildt DE, Kleiman DG, Thompson KV, Baer CK (2010). Wild mammals in captivity: principles and techniques for zoo management..

[B038] Stafford KJ, Spoorenberg J, West DM, Vermunt JJ, Petrie N, Lawoko CRO (1996). The effect of electro-ejaculation on aversive behaviour and plasma cortisol concentration in rams. N Z Vet J.

[B039] Thackare H, Nicholson HD, Whittington K (2006). Oxytocin: its role in male reproduction and new potential therapeutic uses. Hum Reprod.

[B040] Ungerfeld R, Abril-Sánchez S, Toledano-Díaz A, Beracochea F, Castaño C, Giriboni J, Santiaho-Moreno J (2016). Oxytocin administration before sperm collection by transrectal ultrasonic-guided massage of the accessory sex glands in mouflons and bucks. Anim Reprod Sci.

[B041] Ungerfeld R, Casuriaga D, Giriboni J, Freitas-de-Melo A, Silveira P, Brandão FZ (2018). Administration of cloprostenol and oxytocin before electroejaculation in goat bucks reduces the needed amount of electrical stimulation without affecting seminal quality. Theriogenology.

[B042] Voglmayr J (1973). Prostaglandin F2α concentration in genital tract secretions of dairy bulls. Prostaglandins.

[B043] Whittington K, Assinder SJ, Parkinson T, Lapwood KR, Nicholson HD (2001). Function and localization of oxytocin receptors in the reproductive tissue of rams. Reproduction.

[B044] Worley RTS, Nicholson HD, Pickering BT, Courtens JL (1956). Recent progress in cellular endocrinology of the testis..

